# The Landscape of Lyme Borreliosis Surveillance in Europe

**DOI:** 10.1089/vbz.2022.0067

**Published:** 2023-04-12

**Authors:** Archana Nagarajan, Jozica Skufca, Andrew Vyse, Andreas Pilz, Elizabeth Begier, Margarita Riera-Montes, Bradford D. Gessner, James H. Stark

**Affiliations:** ^1^ P95 Epidemiology & Pharmacovigilance, Leuven, Belgium.; ^2^ Vaccines Medical Affairs, Pfizer UK Ltd, Tadworth, United Kingdom.; ^3^ Pfizer Global Medical Affairs, Vaccines, Vienna, Austria.; ^4^ Vaccine Clinical Research and Development, Pfizer Inc, Pearl River, New York, USA.; ^5^ Vaccines Medical Development, Scientific and Clinical Affairs, Pfizer Inc, Collegeville, Pennsylvania, USA.

**Keywords:** Lyme borreliosis, surveillance, Europe, dashboards, vaccine

## Abstract

**Purpose:**

Lyme borreliosis (LB) is the most prevalent tick-borne disease in Europe and the incidence of LB is increasing owing to an expansion in tick habitats. However, LB surveillance is quite heterogeneous across the continent, and for those countries with publicly available data, it is difficult to understand the differences in incidence between countries. The objective of our study was to summarize the publicly available data from surveillance for LB in the form of surveillance reports and/or dashboards and to compare the information available for various countries.

**Methods:**

We identified publicly available LB data (online dashboards and surveillance reports) in the European Union, European Economic Area, the United Kingdom, Russia, and Switzerland.

**Results:**

Of the 36 countries studied, 28 had LB surveillance in place; 23 had surveillance reports, and 10 had dashboards. The dashboards, in general, had more granular data compared with the surveillance reports, but the reports covered longer time periods. LB annual cases, incidence, age, and sex-stratified LB data; manifestations; and regional data were available for most of the countries. LB case definitions varied significantly among the countries.

**Conclusion:**

The study highlights large differences in LB surveillance systems, including representativeness, case definitions, type of data available that might inhibit comparison of data between countries and accurate determination of burden of disease, and risk groups within countries. Standardization of case definitions across countries would be a useful first step enabling comparisons between countries and contribute to recognizing the true burden of LB in Europe.

## Introduction

Lyme borreliosis (LB), the most common tick-borne disease in Europe, is caused by the spirochete complex *Borrelia burgdorferi sensu lato* and transmitted by the bite of *Ixodes* ticks (Stanek et al., 2012). The most common clinical manifestation of LB is a gradually expanding erythematous skin rash known as erythema migrans (EM), which is due to the dissemination of bacteria from the bite site through the skin. If untreated or inadequately treated with antibiotics, the infection can disseminate via the bloodstream to other parts of the body, where it can cause serious manifestations affecting the nervous system, joints, or heart (Kullberg et al., 2020; Lindgren et al., 2006; Marques, 2008).

In Europe, ∼65,000–85,000 LB cases are diagnosed and reported to health officials each year (incidence of 22/100,000 person-years in Western Europe); however, owing to the frequency of undiagnosed LB and inconsistency of case reporting, the true incidence of the disease is underestimated (Sykes and Makiello, 2017).

The underestimated burden of LB in Europe is a reflection of the variation in country-specific public health surveillance systems and reporting practices. Lorenc et al. (2017), reported in detail on the existing surveillance systems for LB in 32 European countries (Lorenc et al., 2017), which provides an overview of the surveillance systems and case definitions for LB in the European Union (EU). Even though the first common European case definitions for LB were developed almost 25 years ago by the European Union Concerted Action on Lyme Borreliosis (EUCALB), and further refined in 2011, they have not been implemented widely for reporting of LB across Europe (Stanek et al., 2011). EUCALB provided clinical case definitions for various LB manifestations and the laboratory and clinical evidence that could support these case definitions (Stanek et al., 2011).

Some countries, such as Austria, have no public health surveillance in place for LB, whereas others, such as Lithuania and Romania, have robust national surveillance (Lorenc et al., 2017). As such, the European Commission in 2018 added Lyme neuroborreliosis (LNB) to the list of diseases under EU epidemiologic surveillance and has released a common LNB case definition (European Center for Disease Prevention and Control, 2018; The Lancet Editorial Board, 2018; Van den Wijngaard et al., 2017).

Public health surveillance of LB (and LNB) and other infectious diseases can help measure the burden of a disease, monitor trends, identify high-risk populations, detect outbreaks of diseases, and guide the planning and implementation of programs for the prevention and control of various diseases (Centers for Disease Control and Prevention, 2001; Groseclose and Buckeridge, 2017; Nsubuga et al., 2006). Public health surveillance for most infectious diseases are designed not to detect all cases, but to provide rapid and timely insights into the changes in disease trends, providing valuable information for policy makers to implement public health interventions, which may include safe and efficacious vaccines.

The National Immunization Technical Advisory Groups (NITAGs), present in most industrialized nations and some developing countries, inform the policy makers who make evidence-based decisions on vaccines. They often use surveillance to assess the burden of disease to recommend any preventive interventions, as well as for cost–benefit analysis, disease modeling, and other purposes (Duclos, 2010; Gessner et al., 2010). These committees also report on the effect of vaccination postlicensure relying on the public health surveillance system to monitor disease burden.

Given the importance of surveillance, this article sought to catalog and characterize publicly available public health surveillance data across Europe that can be used by public health officials and researchers to understand the burden of LB; to study the emerging trends, particularly within a country; and to assess potential benefits with the introduction of safe and efficacious vaccines and public health interventions in the future. Furthermore, this study provides a framework for interpreting the data reported from these systems.

## Methods

To evaluate LB public health surveillance systems in Europe, we searched 36 countries in the EU and the European Economic Area (EEA) plus Switzerland, Russia, Turkey, and the United Kingdom (Table 1). We first identified the countries with LB surveillance in place and then sought to identify the national public health institutes/agencies responsible for infectious disease surveillance in the countries of interest through an online search. We then performed a search of the public health institute websites to find the online dashboards (visualization tools that integrate, analyze, and display data related to the spread of a disease) and surveillance reports containing LB data. The search was limited to the data reported between 2005 and 2020. In some cases, the public health agency was contacted directly to get information on the availability of dashboards (public health agencies from the Czech Republic, Estonia, Finland, Denmark, Latvia, Lithuania, Norway, Poland, Slovakia, and Slovenia).

**Table 1.** Characteristics of Surveillance Systems on Lyme Borreliosis in Europe

No.	Country	LB statutorily notifiable	Other LB surveillance in place	Notifiable since year	National coverage of surveillance system	LB manifestation reported	Surveillance system and/or case definitions used
European Union, EEA, Russia, and Switzerland							
1	Austria	N	N	NA	N	NA	NA
2	Belgium	N	Y	NA	Y	NA	Epidemiologic surveillance of LB through a network of laboratories, GPs, and National Reference Centre for *Borrelia burgdorferi*. The sentinel laboratory network reports the number of positive serological tests (weekly), and the data are published in the reports. No laboratory test is needed for the diagnosis of EM, and clinical information for the cases is not recorded.
3	Bulgaria	Y	NA	2008	Y	LB and LNB	Clinician-reported and laboratory-confirmed. ECDC case definition. All reported cases are laboratory-confirmed except some EM cases, which are clinically diagnosed.
4	Croatia	Y	NA	1991	Y	LB	Clinician-reported and laboratory-confirmed.
5	Cyprus	N	N	NA	N	NA	NA
6	Czech Republic	Y	Y	1986	Y	LB (from 2005)	LB reported by clinicians to district health officers, who complete the standardized case reports and direct interviews with patients. District public health officers enter the case reports into an electronic database and transfer them weekly to the regional public health offices. Regional officers forward the data to the NIPH.
7	Denmark	Y	NA	1991	Y	LNB	Only LNB is reported by physicians and laboratories.
8	Estonia	Y	NA	1992	Y	LB (ICD-10, A69.2)	EM is reported by physicians, confirmed by laboratories.
9	Finland	Y	NA	1995	Y	EM+LB	Physicians report EM to Register for Primary Health Care Visits (Avohilmo) and laboratories report serologic or molecular confirmation of LB to the National Infectious Diseases Register.
10	France	N	Y	2009	Y	EM, LNB, carditis, ACA, LA	Voluntary (sentinel) reporting by physicians on clinical EM (not laboratory-confirmed), LNB, or other disseminated manifestations (requires confirmation by ELISA and Western blot). Network of ∼500 GPs and pediatricians equally distributed across French territories.
11	Germany	Y in 9/16 states	NA	2013	N (in 9/16 states)	EM, LNB, and LA	Surveillance of LB is a state responsibility: each state has its own system and related laws. LB, notified to the RKI, if patient has any of the three manifestations: EM, acute LNB, or LA. EM is only clinically diagnosed; LNB and LA are also laboratory-confirmed.
12	Greece	N	N	NA	N	NA	NA
13	Hungary	Y	NA	1998	Y	EM	EM reported by physicians. They fill out infectious disease report card and report to the National Center of Epidemiology; recorded in the National Database of Epidemiological Surveillance System.
14	Iceland	N	Y	NA	Y	NA	LB cases are based on automatic notifications from the electronic medical record system (Saga) without the accompanying clinical information or laboratory-confirmation.
15	Ireland	Y	NA	2012	Y	LNB	Physicians and laboratories report cases to the Health Protection Surveillance Centre.
16	Italy	N	NA	NA	N	NA	NA
17	Latvia	Y	NA	1997	Y	LB	LB notified by physicians, but there is a lack of case definition. Reported to the Latvian Center for Disease Control.
18	Lithuania	Y	NA	1991	Y	LB (ICD-10, A69.2)	LB notified by physicians and/or laboratory-confirmed. Reported to the Lithuanian National Public Health Centre under the Ministry of Health.
19	Liechtenstein	N	NA	NA	N	NA	NA
20	Luxembourg	Y	NA	2019	Y	LB	LB notified by physicians and laboratory-confirmed.
21	Malta	N	N	NA	N	NA	NA
22	The Netherlands	N	Y	NA	N	NA	No obligation to report LB, however, clinicians are required to maintain records; however, recording of LB varies because there is no single, recognized definition. The National Institute of Environment and Health also encourages those who have been bitten by a tick or have contracted LB to report on the tick radar website.
23	Norway	Y	NA	1995	Y	EM and disseminated LB	LB reported by physicians and laboratory-confirmed. LB reportable by statute includes only laboratory-confirmed (not early localized EM) and is reported to MSIS.
24	Poland	Y	NA	1998	Y	LB (EM, LA, LNB, other)	LB notified by physicians and laboratories. EM, LNB, and other disseminated forms are reported to the district public authority, which completes the standardized case reports and directs an interview with the patient. Regional officers send aggregated reports to the NIPH—National Institute of Hygiene in Warsaw.
25	Portugal	Y	NA	1999	Y	EM and disseminated LB	LB notified by physicians to health authority, Direcção Geral de Saúde. EM and late manifestations with laboratory confirmation.
26	Romania	Y	NA	2007	Y	EM, LNB, and other manifestations	LB notified by physicians and laboratory-confirmed. There are specific criteria for acceptance of laboratory reports. Reported to district health officials by physicians and laboratories, and they report to the National Institute of Public Health.
27	Russia	Y	NA	Not clear	Y	EM and other manifestations	EM notified by physicians and other forms are laboratory-confirmed.
28	Slovakia	Y	NA	1987	Y	ICD-10 codes: EM (A69.2); LNB (G63.0); LA (M01.2)	LB reportable by statute includes only laboratory-confirmed LB and is reportable to the National Surveillance System.
29	Slovenia	Y	NA	1986	Y	ICD-10 codes: EM (A69.2); Polyneuropathy (G63.0); LA (M01.2), Meningitis (G01.0)	LB is reportable by all physicians and laboratories. LB reportable by statute includes EM and various disseminated forms of LB and is reported to the National Institute of Public Health.
30	Spain	Y, in some regions	NA	Varies	N	Region dependent	Surveillance of LB is a subnational responsibility: each region has its own system and related laws. LB is reportable by statute only in some regions (autonomous communities), reported by physicians and laboratories on EM without laboratory or laboratory-confirmed LB.
31	Sweden	N	N	NA	N	NA	NA
32	Switzerland	N	Y	2008	Y	EM and other manifestations	Data are collected using a voluntary system of physicians, called Sentinella. Physicians report tick-bite consultations and also LB with clinical symptoms and laboratory confirmation and when antibiotic treatment is initiated.
33	Turkey	N	N	NA	N	NA	NA
United Kingdom							
34	England and Wales	N (by GPs) and Y (by laboratories)	N	2010 (laboratories)	Y	LB (laboratory-confirmed)	Official surveillance is based on reporting laboratory-confirmed cases only. LB is monitored in England and Wales through routine surveillance. Data are published in the quarterly Health Protection Report and annually in the UK Zoonoses Report. Medical practitioners in England and Wales are not required to report cases of LB.
35	Scotland	N (by GPs) and Y (by laboratories)	N	2010	Y	LB (laboratory-confirmed)	Obligatory reporting of positive laboratory test results. Tests are performed at the National Lyme Borreliosis Testing Laboratory, Raigmore Hospital, Inverness.
36	Northern Ireland	N	Y	2010	Y	LB (laboratory-confirmed)	Cases of LB are not reportable by statute by medical practitioners. Routine data include only positive laboratory test results; however, in contrast to England, Wales, and Scotland, where reporting is mandatory, here cases are reported voluntarily by laboratories.

ACA, acrodermatitis chronica atrophicans; ECDC, European Center for Disease Control; EEA, European Economic Area; ELISA, enzyme-linked immunosorbent assay; EM, erythema migrans; GP, general practitioner; ICD-10, *International Classification of Diseases, Tenth Revision*; LA, Lyme arthritis; LB, Lyme borreliosis; LNB, Lyme neuroborreliosis; MSIS, Norwegian Surveillance System for Communicable Diseases; N, no; NA, not applicable; NIPH, National Institute of Public Health in Prague; RKI, Robert Koch Institute; Y, yes.

The searches were all performed using Google Chrome^®^, which translated the web pages from local European languages to English. Furthermore, we collected information on country-specific case definitions for LB, the manifestations reported, time period for surveillance, geographic stratifications, and the type of surveillance system used in countries that reported LB. Whenever the information on case definitions was not available, we directly contacted the public health institutes of the respective countries to obtain the information (*e.g*., the Bulgarian National Centre for Infectious and Parasitic Diseases). We also used DeepL Pro for translation of surveillance reports whenever language support was needed.

Although some countries report information on ticks in the form of dashboards or in surveillance reports, for the purpose of this study, we have restricted the scope to publicly available surveillance information describing LB in humans. Because all data collected for this study were publicly available surveillance data and no patient consent was involved, Institutional Review Board approval was waived.

## Results

Of the 36 countries searched online, 28 had public health surveillance for LB. The eight countries with no identified public health surveillance system for LB were Austria, Cyprus, Greece, Italy, Liechtenstein, Malta, Sweden, and Turkey. Twenty-three countries reported the cases and/or incidence and other demographics through surveillance reports, and nine countries presented data as dashboards from which data could be downloaded and these countries also produce surveillance reports. Denmark presented the surveillance data through the dashboard, but does not publish surveillance reports. Four countries with LB surveillance—Spain, Luxembourg, the Netherlands, and Iceland—have neither reports nor dashboards available to disseminate the LB data publicly.

### Surveillance systems in Europe

**FIG. 1.**
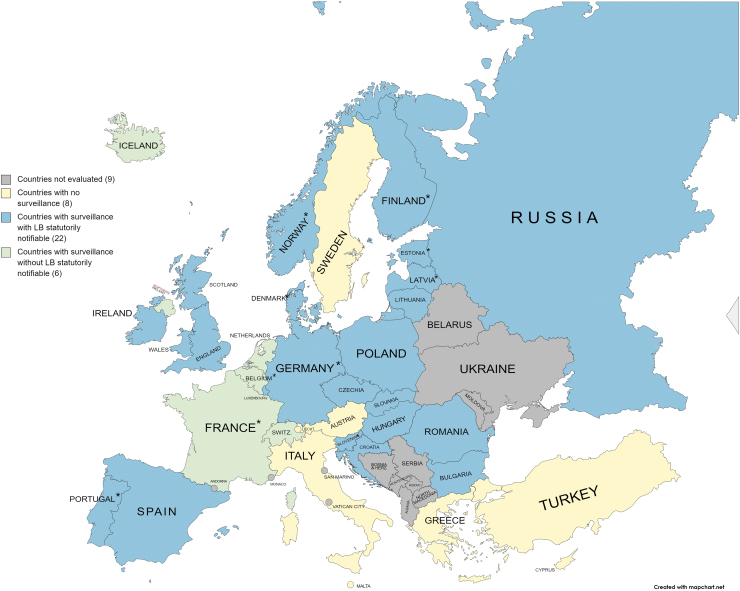
Summary description of countries assessed for LB surveillance. European countries with no LB surveillance (*yellow*), LB surveillance present with LB reported by statute (*blue*), and LB surveillance present without LB statutory reporting (*green*). *Countries with dashboards. LB, Lyme borreliosis; LNB, Lyme neuroborreliosis. The map depicted in the figure was created using mapchart.net and was adapted by the authors.

LB is statutorily reportable in 22 out of the 28 countries with LB surveillance (Table 1 and Fig. 1). Out of these 22 countries, 20 have national surveillance in place; Germany and Spain have only regional surveillance limited to certain states. Of the six countries where LB surveillance is in place but not statutorily reportable, Belgium, France, and Switzerland have a sentinel surveillance system in which a group of general practitioners (GPs) and/or laboratories, representative of various regions of the country, voluntarily report cases of LB; in Northern Ireland, positive laboratory cases are voluntarily reported to national authorities; Iceland has surveillance with automated reporting through the electronic health records without the accompanying clinical or laboratory information; in the Netherlands, GPs are required to maintain a record of LB consultations (Table 1).

### Case definitions and reporting of LB

Table 1 summarizes the case definitions used by the 28 countries that have public health surveillance for LB.

Denmark and Ireland report only cases of LNB. In some countries including the Czech Republic, Hungary, Finland, Germany and Slovenia, EM is reported by clinicians, and laboratory confirmation is not required. For early- and late-disseminated manifestations, laboratory tests are needed to confirm the diagnosis in most countries (*e.g*., Bulgaria, Croatia, Estonia, Finland, Latvia, Lithuania, Luxembourg, Norway, Poland, Portugal, Romania, Slovakia, and Slovenia). The United Kingdom (except Northern Ireland) and Slovakia have only mandatory reporting of confirmed cases by laboratories, and GPs do not report EM. France and Poland report LB based on EUCALB case definition recommendations (Stanek et al., 2011). However, most countries do not follow the EUCALB case definitions, in which each clinical manifestation has a clinical case definition and accompanying laboratory evidence to support the diagnosis.

The countries with nonmandatory sentinel surveillance (*i.e*., Belgium, France, and Switzerland) also vary in their surveillance mechanisms and reporting. In Belgium, a sentinel network of laboratories notifies the positive serological cases, and GPs report EM without laboratory confirmation. The national reference laboratory reports the strains of *Borrelia* for the cases sent to them. In France, GPs notify EM without laboratory confirmation, but late manifestations (*e.g*., LNB, Lyme arthritis, carditis) require laboratory confirmation with both enzyme-linked immunosorbent assay and Western blot testing. Switzerland has a network of GPs that report tick bite consultations and LB with clinical symptoms that are laboratory confirmed.

### Data available from dashboards

Ten countries have LB data accessible in the form of dashboards: Belgium, Denmark, Estonia, Finland, France, Germany, Latvia, Norway, Portugal, and Slovenia (Fig. 1 and Table 2). Seven countries have >1 dashboard (Table 3). For example, Estonia has two dashboards, one reporting LB as part of the tick-borne disease statistics and another presenting additional health statistics maintained by the National Institute for Health Development. Most often the various databases represent different sources from which the data are obtained including GP reporting of LB cases without laboratory confirmation, laboratory reporting of laboratory-confirmed cases, and hospitalizations. Table 3 summarizes information regarding access to databases (web address), the public health authority responsible for the maintenance of the dashboards, and, in case of countries with >1 database, the type of data that can be obtained from the databases.

**Table 2.** Characteristics of Publicly Available Data on Lyme Borreliosis in Europe

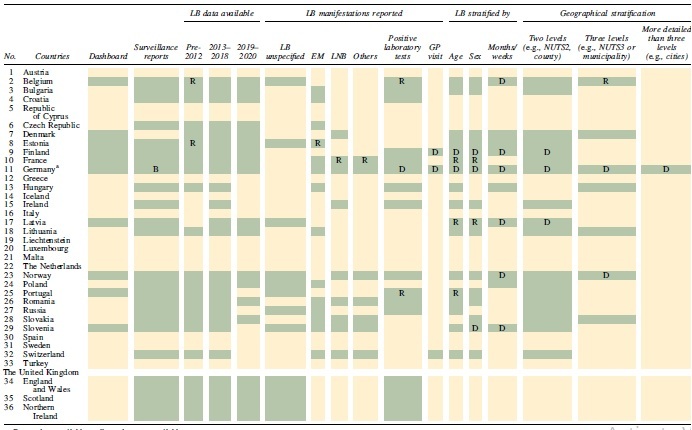

Green, data available; yellow, data not available.

^a^ Only 9/16 German states report LB by statute.

B, German surveillance reports are available only for Bavaria; D, dashboard only; EM, erythema migrans; GP, general practitioner; LB, Lyme borreliosis; LNB, Lyme neuroborreliosis; NUTS, nomenclature of territorial units for statistics; R, surveillance report only.

**Table 3.** Information on the Dashboards Available (in 10 of 36 Countries)

Countries	Dashboard, n	Body responsible for collating surveillance data	Database name	URL	Further information
Belgium	1	Sciensano	EPISTAT Belgian Infectious Diseases	https://epistat.wiv-isp.be/dashboard	*Borrelia burgdorferi* (Lyme) consultations and/or laboratory tests
Denmark	1	SSI	SSI online database	https://statistik.ssi.dk//sygdomsdata#!/?sygdomskode=NEBO&xaxis=Aar&show=Graph&datatype=Individual	LNB reported by physicians
	2	SSI	SSI online database	https://statistik.ssi.dk/sygdomsdata#!/?sygdomskode=NEBOM&xaxis=Aar&show=Graph&datatype=Laboratory	LNB reported by laboratories
Estonia	1	Terviseamet/Republic of Estonia Health Board	Puugihaiguste statistika (Tick disease statistics)	https://www.terviseamet.ee/et/nakkushaigused/tervishoiutootajale/nakkushaigustesse-haigestumine/puugihaigused	LB
	2	Tervise Arengu Instituut./National Institute for Health Development	The Health Statistics and Health Research Database	https://statistika.tai.ee/pxweb/en/Andmebaas/Andmebaas__02Haigestumus__02Nakkushaigused/NH02.px	LB (ICD-10 code, A69.2)
Finland	1	THL	National infectious register (TTR-NIDR)	https://sampo.thl.fi/pivot/prod/fi/ttr/shp/fact_shp?row=area-12260&column=time-12059&filter=reportgroup-12465	Microbiologically confirmed LB cases reported by laboratories (primarily representing disseminated LB)
	2	THL	Register for Primary Health Care Visits (Avohilmo)	https://sampo.thl.fi/pivot/prod/en/infestat/borre/fact_infestat_borre?row=hcdmunicipality2020-572572&column=weeks-546468&filter=measure-546834	LB (ICD-10 code A69.2) in primary health care (primarily representing EM)
France	1	Sentinelles Network	Sentinelles	https://www.sentiweb.fr/france/fr/?page=maladies&mal=18	Laboratory-confirmed LB
Germany	1	RKI	SurvStat@RKI2.0	https://survstat.rki.de/Content/Query/Create.aspx	Case of LB, which is reported to the RKI, is any of the three following manifestations: (1) clinically diagnosed EM (ICD-10 code, A69.2); (2) laboratory-confirmed LA (ICD-10 code, M01.2); and (3) laboratory-confirmed acute neuroborreliosis (ICD-10 code, G01.-)
	2	GBE-BUND	Information System of the Federal Health—Monitoring Diagnostic data of the hospitals	https://www.gbe-bund.de/gbe/pkg_isgbe5.prc_menu_olap?p_uid=gast&p_aid=37717048&p_sprache=E&p_help=3&p_indnr=550&p_indsp=&p_ityp=H&p_fid=	LB (ICD-10 code, A69.2), arthritis in LB for hospitalizations (ICD-10 code, M01.2)
Latvia	1	SPKC/Latvian Centre for Disease Prevention and Control	Veselības statistikas datubāze (Health Statistics Database)	https://statistika.spkc.gov.lv/pxweb/lv/Health/Health__Saslimstiba_Slimibu_Izplatiba__Infekcijas_un_parazitaras_slimibas/INF010_Saslimstiba_ar_inf_kopa_regioni.px	Incidence of infectious diseases by regions. LB (ICD-10 code, A69.2)
	2	SPKC/Latvian Centre for Disease Prevention and Control	Veselības statistikas datubāze (Health Statistics Database)	https://statistika.spkc.gov.lv/pxweb/lv/Health/Health__Saslimstiba_Slimibu_Izplatiba__Infekcijas_un_parazitaras_slimibas/INF020_Infekcijas%20slimibas%20pa%20menesiem.px	Reportable communicable diseases (monthly data). LB (ICD-10 code, A69.2)
Norway	1	FHI/National Public Health Institute Norway	MSIS	http://msis.no	Laboratory-confirmed LB data
	2	Helsedirektoratet/Directorate of Health	NRPHC/KPR	https://statistikk.helsedirektoratet.no/bi/Dashboard/072ab7b5-ca53-48a0-8f5f-e9a687a150f7?e=false&vo=viewonly	Mandatory registry for municipal/primary health and care services, the NRPHC was established in December 2017, uses ICPC-2 code, A78 referring to few infections, including LB; however, LB is not separately reported
	3	Helsedirektoratet/Directorate of Health	NPR/hospitalization register	https://www.helsedirektoratet.no/statistikk/statistikk-fra-npr/aktivitet-somatiske-sykehus	Hospital visits due to LB (ICD-10 code, A69.2), LA (ICD-10 code, M01.2), LNB (ICD-10 code, G01.9)
Portugal	1	Institutos Nacionais de Estatística de Portugal/National Institute of Statistics	Casos notificados de doenças de declaração obrigatória/Mandatory notification Diseases	https://www.ine.pt/xportal/xmain?xpid=INE&xpgid=ine_indicadores&indOcorrCod=0008458&selTab=tab0&xlang=pt	LB reported by physicians and late manifestations are laboratory-confirmed
Slovenia	1	NIJZ	NIJZ Podatkovni portal—Notifiable infectious diseases	https://podatki.nijz.si/Search.aspx?searchquery=Lymska&px_language=sl&px_db=NIJZ%20podatkovni%20portal&rxid=99e36e86-4a38-4df5-b6f0-3849f420c2f0	Contains the following data from GPs and laboratory confirmed: LB (ICD-10 code, A69.2), LM (ICD-10 code, G01.0), Lyme polyneuropathy (ICD-10 code, G63.0), LA (ICD-10 code, M01.2)
	2	NIJZ	NIJZ Podatkovni portal—Hospital treatment	https://podatki.nijz.si/Search.aspx?searchquery=Lymska&px_language=sl&px_db=NIJZ%20podatkovni%20portal&rxid=99e36e86-4a38-4df5-b6f0-3849f420c2f0	LB (ICD-10 code, A69.2) and LA (ICD-10 code, M01.2) for hospital visits

EM, erythema migrans; FHI, Folkehelseinstituttet [National Public Health Institute Norway]; GBE-BUND, Die Gesundheitsberichterstattung des Bundes [Federal Health Monitoring]; GP, general practitioner; ICPC, *International Classification of Primary Care*; LA, Lyme arthritis; LB, Lyme borreliosis; LNB, Lyme neuroborreliosis; NIJZ, Nacionalni institut za javno zdravje [National Institute for Public Health Slovenia]; NPR, Norsk pasientregister [Statistics from the Norwegian Patient Registry]; NRPHC/KPR, Norwegian Registry for Primary Health Care; SPKC, Slimību profilakses un kontroles centrs [Latvian Centre for Disease Prevention and Control]; SSI, Statens Serum Institut; THL, Terveyden ja Hyvinvoinnin Laitos [Finnish Institute for Health and Welfare].

In general, the publicly available dashboards have more granular data compared with the surveillance reports and report the overall LB cases and/or incidences per year, different levels of geographic stratification (nomenclature of territorial units for statistics NUTS2 and NUTS3 regions and, in some cases, the hospital districts), age, sex, and LB manifestation. Some of them also report seasonality and provide monthly or weekly data (Table 2).

### Data available from surveillance reports

Twenty-three countries have publicly available annual surveillance reports that can be downloaded from the internet (Table 4). The reports are in English or the national language. Table 4 also provides the information on the public health agencies responsible for regular publication of surveillance reports.

**Table 4.** Information on the Surveillance Reports Available (in 23 of 36 Countries)

Countries	Body responsible for collating surveillance reports	URL	Reporting language
Belgium	Sciensano	https://nrchm.wiv-isp.be/fr/centres_ref_labo/borrelia_burgdorferi_Lyme_disease/Rapports/Forms/AllItems.aspx	Separate reports from laboratory sentinel network (laboratoires vigies) and CNR until 2010. From 2011, combined reports. Reports in French.
Bulgaria	National Center of Infectious and Parasitic Diseases	https://www.ncipd.org/index.php?option=com_k2&view=item&layout=item&id=84&Itemid=1337&lang=bg	Reports in Bulgarian.
Croatia	Hrvatski Zavod Za Javno Zdravstvo/Croatian Institute of Public Health	https://www.hzjz.hr/tag/zdravstveno-statisticki-ljetopis	Reports in Croatian.
Czech Republic	SZU/The National Institute of Public Health	www.szu.cz/national-reference-laboratory-for-lyme-borreliosis?lang = 2	Reports in Czech.
Estonia	Republic of Estonia: Health Board (Terviseamet)	https://www.terviseamet.ee/et/nakkushaigused-menuu/tervishoiutootajale/nakkushaigustesse-haigestumine#Nakkushaigustesse%20haigestumine%20Eestis%20alates%202000.%20aastast%20kuude%20ja%20maakondade%20kaupa	Annual reports in Estonian. Infectious disease reports every 4 years in English.
Finland	THL	https://sampo.thl.fi/pivot/prod/fi/infestat/borre/fact_infestat_borre?row=weeks-186790&column=ttrage-186467&filter=measure-322099	Annual reports in English.
France	Sentinelles Network	https://www.sentiweb.fr/france/fr/?page=maladies&mal=18	Reports in French since 2009.
Germany	No one central authority. The local state authorities as applicable for each state.	NA	Annual reports are available only intermittently and for the states of Bayern and Berlin. Others are reported and available only in dashboard.
Hungary	National Center for Epidemiology	www.oek.hu/oek.web?nid=509&pid=3&to=,2475,2465&lang=hun	Reports in Hungarian.
Ireland	Health Protection Surveillance Centre	https://www.hpsc.ie/a-z/vectorborne/lymedisease/epidemiologicaldata	Reports in English available from 2012. Only LNB reported.
Latvia	Latvian Centre for Disease Prevention and Control	https://www.spkc.gov.lv/lv/infekcijas-slimibas-statistika-un-petijumi	Annual reports available in Latvian from 2009.
Lithuania	ULAC	www.ulac.lt/ataskaitos#2013	Annual reports in Lithuanian.
Norway	Norwegian Institute of Public Health	www.msis.no https://www.fhi.no/sys/sok/?type=con-32/cat-750,&term=Lyme#main	Reports in Norwegian.
Poland	NIPH-NIH	http://wwwold.pzh.gov.pl/oldpage/epimeld/index_p.html#uu	Annual reports in Polish.
Portugal	National Institute of Statistics	https://www.ine.pt/xportal/xmain?xpid=INE&xpgid=ine_main	Annual reports in Portuguese.
Romania	National Institute of Public Health	www.cnscbt.ro	Annual reports in Romanian from 2008.
Russia	Federal Service for Supervision of Consumer Rights Protection and Human Welfare	https://www.rospotrebnadzor.ru	Annual report in Russian from 2009.
Slovakia	Public Health Authority of the Slovak Republic	https://www.uvzsr.sk/index.php?option=com_content&view=category&id=25:vyrona-sprava&layout=blog&Itemid=34&layout=default	Annual reports since 2005 in Slovakian.
Slovenia	National Institute of Public Health	https://www.nijz.si/en	Annual reports in Slovenian.
Switzerland	The Sentinella Network	www.sentinella.ch/fr/publications	Reports in French and German since 2008.
The United Kingdom			
England and Wales	Public Health England and Department of Environment, Food and Rural Affairs	https://www.gov.uk/government/publications/zoonoses-uk-annual-reports	Annual reports in English.
Scotland	Public Health Scotland	https://www.hps.scot.nhs.uk/data/latest-epidemiology-reports	Annual reports in English.
Northern Ireland	Department for Environment, Food and Rural Affairs	https://www.gov.uk/government/publications/zoonoses-uk-annual-reports	Annual reports in English.

CNR, Centres de reference; NIPH-NIH, National Institute of Public Health-National Institute of Hygiene; SZU, Státní zdravotní ústav; THL, Finnish Institute for Health and Welfare; ULAC, Centre for Communicable Diseases and AIDS.

In general, the data from surveillance reports are available for more countries and for longer time periods than the dashboards. The reports provide information on cases and incidence of LB and regional and age-specific data. They also report various manifestations of the disease according to the case definitions for each country (Table 2).

## Discussion

With the increase in LB cases and expansion of tick habitats in Europe, possibly because of climate change, it has become increasingly important that European countries have appropriate surveillance systems in place for monitoring LB (Comstedt et al., 2017; Gomes-Solecki et al., 2019; Lindgren et al., 2006; Schiller et al., 2021). The results from this analysis indicated that significant variation in reporting exists across Europe, including the type and case definitions of LB reported (Table 1 and Fig. 1). The limited capabilities within the countries to estimate the true burden of disease, a reflection of underreporting and underascertainment, and the inability to estimate the burden of disease across Europe using surveillance data present significant challenges to introducing and implementing wide-scale public health interventions.

However, at an individual country level, LB surveillance data can provide valuable insights on the trends in LB cases over the years and on the regions that have higher burden of disease. Ten countries offer online dashboards to provide real-time assessments of LB epidemiology and, although limited in number, these countries enable robust and often geographically granular analyses compared with annual surveillance reports.

A country's disease surveillance system can help inform the advice provided by NITAGs to governments regarding vaccine recommendations and public health interventions based on the known burden of disease and populations at risk (Groseclose and Buckeridge, 2017). Hence, the type of surveillance in place (comprehensive versus sentinel, national versus regional) and its coverage may affect these decisions. A cross-sectional survey conducted in 2009 in 28 European countries on tick-born encephalitis (TBE) surveillance and vaccine recommendations found that more robust surveillance of TBE is needed to improve the quality of vaccine recommendations (Stefanoff et al., 2011).

Several of the European countries (*e.g*., Belgium, Lithuania) and several German states have very granular geographic and demographic LB data available, and these can support in describing the burden of disease and risk factors. This is especially important for LB because its presence within a country is affected by the geographic distribution of ticks and *Borrelia* spp. and by differences in geographic and climatic factors and human activities (Van den Wijngaard et al., 2017). Comparison of trends in LB cases among countries over time could provide important information on the burden of disease not only on a national level but in certain European regions as well (*e.g*., Scandinavia and the Baltic countries).

Dashboards offer interactive dynamic data that epidemiologists can use to explore trends and regional patterns more easily than the traditional reports and spreadsheets (Dixon et al., 2021). The COVID-19 dashboards during the current pandemic have been valuable in providing real-time updates and have helped governments make timely decisions with respect to testing, social distancing, and vaccination (Pietz et al., 2020). Nevertheless, country variation in reporting led to challenges in compiling data and comparing between countries. The LB dashboards available in some countries (*e.g*., Belgium, Finland, Denmark, Germany, and Norway) are updated more often and provide information on real-time LB monitoring. They also provide very granular geographic data, such as at the town or city level, that are more detailed than, for example, the NUTS3 regions.

Thus, LB dashboards provide excellent opportunities to understand seasonality and evolving LB situations in various regions within a country, and to identify focal endemic regions, which can help with the decision-making process on public health interventions. However, the absence of LB data for regions within certain countries (*e.g*., Germany has no LB reporting in seven states) can make informed decision-making with respect to overall national health interventions and vaccine recommendations very challenging.

EUCALB recommendations on case definitions for various LB manifestations are followed only by Bulgaria, Slovenia, and Poland. EUCALB provided a common framework on case definitions and laboratory testing required to confirm the manifestations. It has been more than a decade since development, and these definitions have not been adopted by all the European countries. Adoption of these definitions can make the LB surveillance across these countries more robust and comparable, thus describing the true burden of disease in Europe.

### Limitations

Although the publicly available data on LB are an invaluable resource, it is possible to have missed some levels of data available to the public owing to the unavailability of English language websites for most European countries. Furthermore, because of the structure of reporting systems (*e.g*., >1 database being maintained by some countries [Finland, Slovenia]), we might have some challenges with respect to the type of data that each dashboard reports and reconciling them. Surveillance systems generally underreport, and they are not an ideal tool to look at the absolute burden of disease (Allen et al., 2016; World Health Organization, 2000). Furthermore, surveillance systems are dynamic and evolve over time, and the snapshots may therefore not reflect the current environment. Despite these limitations, surveillance data provide an invaluable source to help researchers and policy makers identify trends within a country and implement and monitor appropriate public health interventions.

### Strengths

Previous studies have reported the types of LB surveillance systems that are prevalent in Europe along with the case definitions used and some of the solutions to implement common case definitions (Lorenc et al., 2017; Van den Wijngaard et al., 2017). This study provides additional information on the availability of publicly available data for LB in Europe, providing information on where to find the relevant data in the form of dashboards and/or reports. We have further compared the surveillance systems and case definitions for 36 European countries and the levels of data available for these countries. This work reiterates the need for common case definitions as outlined by EUCALB and other researchers (Stanek et al., 2011; Van den Wijngaard et al., 2017) and the need for the harmonization of surveillance systems across Europe.

## Conclusions

The surveillance of LB in Europe is heterogeneous, and comparison among countries should be done with caution. EUCALB recommendations on case definitions of various LB manifestations can provide a common framework for European countries. This expansion and harmonization of LB surveillance would allow for quantification of the effect of LB on the population and give an accurate assessment for the need for public health interventions, such as potential future vaccination programs.

## Data Availability

All data generated or analyzed during this study are included in this published article.
